# Novel Genetic Variations in Acute Myeloid Leukemia in Pakistani Population

**DOI:** 10.3389/fgene.2020.00560

**Published:** 2020-06-23

**Authors:** Saba Shahid, Muhammad Shakeel, Saima Siddiqui, Shariq Ahmed, Misha Sohail, Ishtiaq Ahmad Khan, Aiysha Abid, Tahir Shamsi

**Affiliations:** ^1^Department of Genomics, National Institute of Blood Diseases and Bone Marrow Transplantation, Karachi, Pakistan; ^2^Jamil-ur-Rahman Center for Genome Research, Dr. Panjwani Center for Molecular Medicine and Drug Research, International Center for Chemical and Biological Sciences, University of Karachi, Karachi, Pakistan; ^3^Department of Hematology, National Institute of Blood Diseases and Bone Marrow Transplantation Karachi, Karachi, Pakistan; ^4^Centre for Human Genetics and Molecular Medicine, Sindh Institute of Urology and Transplantation (SIUT), Karachi, Pakistan

**Keywords:** genomic screening, AML, next generation sequencing, myeloid sequencing panel, novel no-silent somatic mutation

## Abstract

Acute myeloid leukemia (AML) is a hematological malignancy characterized by clonal expansion of blast cells that exhibit great genetic heterogeneity. In this study, we describe the mutational landscape and its clinico-pathological significance in 26 myeloid neoplasm patients from a South Asian population (Pakistan) by using ultra-deep targeted next-generation DNA sequencing of 54 genes (∼5000×) and its subsequent bioinformatics analysis. The data analysis indicated novel non-silent somatic mutational events previously not reported in AML, including nine non-synonymous and one stop-gain mutations. Notably, two recurrent somatic non-synonymous mutations, i.e., *STAG2* (causing p.L526F) and *BCORL1* (p.A400V), were observed in three unrelated cases each. The *BCOR* was found to have three independent non-synonymous somatic mutations in three cases. Further, the *SRSF2* with a protein truncating somatic mutation (p.Q88X) was observed for the first time in AML in this study. The prioritization of germline mutations with ClinVar, SIFT, Polyphen2, and Combined Annotation Dependent Depletion (CADD) highlighted 18 predicted deleterious/pathogenic mutations, including two recurrent deleterious mutations, i.e., a novel heterozygous non-synonymous SNV in *GATA2* (p.T358P) and a frameshift insertion in *NPM1* (p.L258fs), found in two unrelated cases each. The *WT1* was observed with three independent potential detrimental germline mutations in three different cases. Collectively, non-silent somatic and/or germline mutations were observed in 23 (88.46%) of the cases (0.92 mutation per case). Furthermore, the pharmGKB database exploration showed a missense SNV rs1042522 in *TP53*, exhibiting decreased response to anti-cancer drugs, in 19 (73%) of the cases. This genomic profiling of AML provides deep insight into the disease pathophysiology. Identification of pharmacogenomics markers will help to adopt personalized approach for the management of AML patients in Pakistan.

## Introduction

Acute myeloid leukemia (AML) is the most frequent form of acute leukemia in adults with a poor survival rate of about 5 years only (Horton and Huntly, 2012; Cancer Genome Atlas Research Network et al., 2013). It is caused by pathogenic variations in normal progenitor myeloid hematopoietic cells, leading to altered differentiation, proliferation, and self-renewal capability of the cells (Papaemmanuil et al., 2016). In the last decade, there has been significant increase in understanding of underlying mutational landscape of AML (Arber et al., 2016; Papaemmanuil et al., 2016). Consequently, the prognosis, diagnosis, and treatment have been transformed from histological findings to cytogenetic and genomic testing (Grimwade et al., 2010). Analyzing the genetic alterations in AML can be helpful to reduce ambiguities in further characterization of the molecular heterogeneity of normal karyotype AML (Renneville et al., 2008).

Recent studies on the molecular pathogenesis have identified prognostic significance of genetic variation and their contribution in the pathogenesis of AML (Papaemmanuil et al., 2016). The improved AML prognosis associated with mutated *NPM1* and biallelic mutations in the *CEBPA* have resulted in a change in the disease definition (May Green et al., 2010; Hollink et al., 2011). These recent advances have changed the classification and introduced molecular subtypes of the AML with gene mutations (*NPM1* and *CEPBA*) by the recommendation of WHO classification of hematopoietic tumors in 2008 (Campo et al., 2008). In the revised version of 2016, WHO classification introduced additional germline predisposition associated with genetic alterations in the genes *CEBPA*, *DDX41*, *RUNX1*, *ANKRD26*, *ETV6*, and *GATA2* (Cazzola, 2016; Swerdlow et al., 2016). Further studies on genetic landscape of AML have expanded the mutational spectrum where *TET2*, *DNMT3A*, *NPM1*, *SRSF2*, and *ASXL1* genes are mutated frequently in elderly people (Prassek et al., 2018).

Mutational profiling plays an important role in the diagnosis of AML and is now routinely available as a part of the diagnostic workup. It provides diagnostic accuracy, which increases the precision in risk stratification and helps in adopting therapeutic options (Papaemmanuil et al., 2013; Kuo and Dong, 2015). The development of *FLT3* and *IDH2* inhibitors (Lee et al., 2017; Stein et al., 2017) is achieved only by extensive genomic studies. With the advent of next-generation DNA sequencing (NGS), the cost of genome sequencing has decreased significantly. Amplicon-based targeted sequencing represents an attractive mutation detection method in selected gene panels (Harismendy et al., 2011; Jünemann et al., 2013). This strategy needs less amount of DNA and provides large data of multiple genes in a short turnaround time. Therefore, the genomic tractability of AML makes it a feasible option for targeted NGS testing clinically. The aim of this study was to assess the frequency and clinico-pathological significance of frequently mutated genes by targeting sequencing in AML cases. The targeted sequencing panel comprises of genes involved in various biological functions such as epigenetic regulator genes, the cohesin complex protein encoding genes, genes of activated signaling, tumor repressor genes, and spliceosome genes. This is the first study on molecular characterization of AML patients from South Asia using myeloid sequencing panel, which will be helpful in early diagnosis as well as risk management.

## Materials and Methods

### Ethical and Consent Statement

For this study, 26 AML patients were recruited and sequenced for TruSight myeloid sequencing panel between December 2015 and 2018. These patients included 15 males and 11 females with a median age of 35 years (range: 7–51 years). The clinical presentation of the cases, chromosomal abnormalities, and percentage of circulating blast cells are given in Supplementary Table S1. The study design was approved by the Research Ethics Committee and Review Board of NIBD, and in accordance with the tenets of the Declaration of Helsinki. A written informed consent was obtained from patients and their legal guardians for participation in this study and publication of the findings. Peripheral venous blood specimens of all the recruited patients were collected in EDTA tubes, and stored at 4°C till DNA isolation and subsequent analysis.

### DNA Extraction

Genomic DNA was isolated from peripheral blood by using QIAamp DNA Blood Mini Kit (Qiagen, Hilden, Germany) according to the manufacturer’s protocol. The quality of the extracted DNA was assessed by 2% agarose gel and quantified by Qubit DNA HS Assay Kit (Invitrogen, Thermo Fisher Scientific, United States).

### Myeloid Sequencing Panel

TruSight myeloid sequencing panel (Illumina, San Diego, CA, United States) is designed to sequence targeted regions of 54 genes frequently reported for somatic mutations (complete coding exons of 15 genes and exonic hotspots of 39 genes). The genes whose complete coding exons were sequenced include *BCOR, BCORL1, CDKN2A, CEBPA, CUX1, DNMT3A, ETV6/TEL, EZH2, KDM6A, IKZF1, PHF6, RAD21, RUNX1/AML1, STAG2*, and *ZRSR2*, and exonic hotspots of 39 genes include *ABL1, ASXL1, ATRX, BRAF, CALR, CBL, CBLB, CBLC, CSF3R, FBXW7, FLT3, GATA1, GATA2, GNAS, HRAS, IDH1, IDH2, JAK2, JAK3, KIT, KRAS, KMT2A/MLL, MPL, MYD88, NOTCH1, NPM1, NRAS, PDGFRA, PTEN, PTPN11, SETBP1, SF3B1, SMC1A, SMC3, SRSF2, TET2, TP53, U2AF1*, and *WT1*. The panel consists of 568 amplicons (length range: 225–275 bp) and covers ∼141 kb of genomic region of ∼250-bp fragment lengths.

### DNA Libraries Preparation

The sequencing libraries were prepared from 50 ng of genomic DNA per sample using TruSight myeloid sequencing panel according to the manufacturer’s protocol. Briefly, the libraries were prepared by annealing uniquely targeted specific oligos at upstream and downstream to the region of interest (ROI), followed by the removal of unbound oligos in subsequent washing steps by using a filter plate. In extension and ligation step, DNA polymerase was used to connect the hybridized upstream and downstream oligos resulting in the formation of products containing the targeted regions of interest flanked by sequences required for amplification. Next, the amplification step added indexes adapters and prepared for cluster generation. Then, the libraries were cleaned up by using AMPure XP beads to purify PCR products. After the purification, libraries were quantified by Qubit DNA HS Assay kit (Life Technologies, United States).

Libraries were normalized to attain equal library representation that pooled in batches of four samples as per the given guideline. A Pooled Amplicon Library (PAL) was prepared by mixing 5 μl of each of uniquely indexed library. Then, libraries were diluted by taking 6 μl of PAL and 594 μl of ice-cold HT1 incorporation buffer and heat-denatured at 92°C for 2 min. The diluted amplicon libraries were placed on the ice water bath for 5 min, and then 600 μl of the final sample was loaded into the sequencing reagent cartridge kit V2 (MS-102-2002). Workflow for DNA library preparation using Illumina TruSight myeloid sequencing panel is given in Supplementary Figure S1. The DNA sequencing was performed on a MiSeq instrument with standard V2 flow cells with paired end sequencing (150 bp × 2), as per the manufacturer’s instruction.

### Sanger Sequencing

Sanger Sequencing was performed to confirm the variants that were identified as pathogenic through standard protocol (BigDye^®^ Terminator v3.1 Cycle Sequencing Kit, Applied Biosystems^®^). The status of known mutations in *NPM1* and *FLT3* genes were checked by Sanger sequencing and later by allele-specific polymerase chain reaction (PCR) and PCR-restriction fragment length polymorphism (PCR-RFLP) analysis. Electropherogram of identified mutation in AML cases are given in Supplementary Figure S2.

### Data Analysis

**FIGURE 1:**
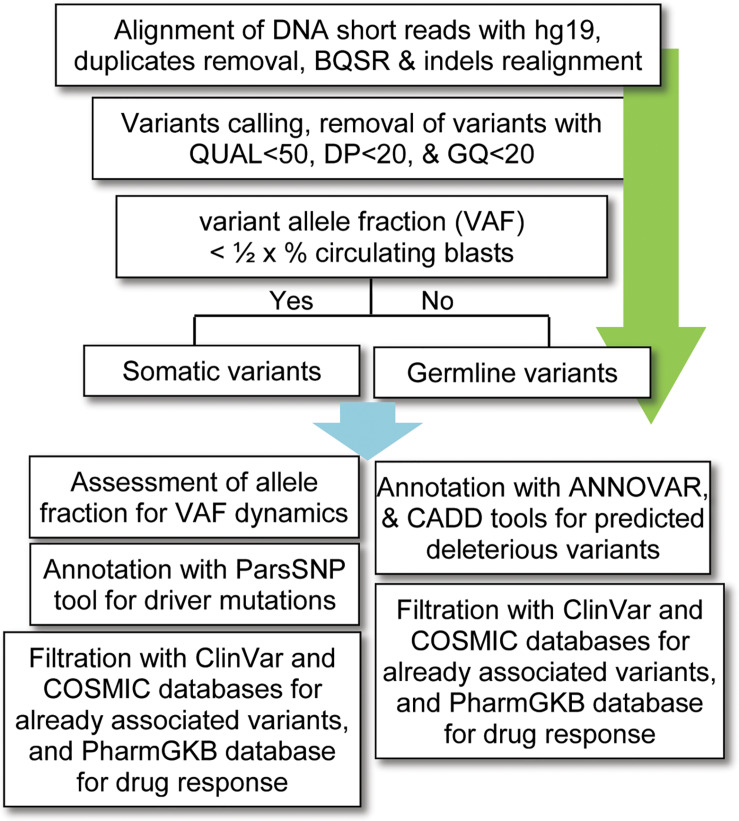
Workflow for bioinformatics analysis of the DNA sequencing data. BQSR, base quality score recalibration, QUAL, variant quality score, DP, depth of coverage, GQ, genotype quality, VAF, variants allele fraction, CADD, combined annotation dependent depletion.

For data analysis, variants calling was performed using the standard pipeline, as described elsewhere (Lek et al., 2016). The alignment of short DNA sequences with human reference genome hg19 (UCSC) was performed by using Burrows–Wheeler Aligner (BWA-MEM) algorithm (Li and Durbin, 2009). The sequence alignment files (SAM) were converted into binary format (BAM) files using SAMtools (Li et al., 2009); and the removal of duplicates (PCR artifacts) was performed using the PICARD tool^1^. The base quality score recalibration (BQSR), realignment around small insertions and deletions, and variants calling were carried out by using on-instrument pipeline with Genome Analysis Tool Kit (GATK) best practices (DePristo et al., 2011). The variants with QUAL < 50, GQ < 20, and population variant allele frequency ≥1% in either gnomAD_genome or 1000 Genomes Project were filtered out as recommended previously (Tyner et al., 2018). Given that no matching normal tissue samples were sequenced, a bit stringent criterion was applied for somatic variants; i.e., the variants with allelic fraction (VAF) less than half of the percent circulating blast cells in each patient (VAF < 1/2 × %circulating blasts) were considered as somatic. To find possibly pathogenic and/or deleterious somatic associated with AML, a multi-tool prioritization approach was adopted, as recommended by American College of Medical Genetics and Genomics (Richards et al., 2015; Figure 1). The identified variants were annotated with ANNOVAR (Yang and Wang, 2015) and Variants Effect Predictor (McLaren et al., 2016) tools to determine their functional consequences. The deleterious impact of non-synonymous variants was assessed with SIFT, Polyphen2, and Combined Annotation Dependent Depletion (CADD), as described previously (Shakeel et al., 2018).

To prioritize biologically active driver mutations over inactive passengers, the parsimony-guided unsupervised functional impact predictor ParsSNP tool was used. This tool uses an expectation maximization framework to find mutations that explain tumor incidence broadly, without using predefined training labels that can introduce biases (Kumar et al., 2016). The identified variants were also searched in ClinVar database (Landrum et al., 2014) for pathogenic/likely pathogenic association with myeloid malignancies. The interaction between the proteins with deleterious variants in the same samples was determined using STRING database (von Mering et al., 2005). The curation from pharmGKB database (Hewett et al., 2002) was performed to determine variants that likely have a role in leukemic chemotherapy.

## Results

This study involves determination and assessment of genetic variations in 26 AML cases of a South Asian population (Pakistan) through Illumina TruSight myeloid sequencing panel. This panel was designed to identify somatic mutations in myeloid malignancies. The median depth of coverage for coding variants was 4979×, and average coverage was 15,477×. Likewise, for non-coding regions, the median depth of coverage was 9558× and average coverage was 24,348×. After filtering out the variants with QUAL < 50, DP < 20, and GQ < 20, there were 293 variants in 54 genes, where each patient contained on average 80 variants (SD ± 8.5). The variants allele fraction distribution revealed the median of 0.51 across all 26 samples (Supplementary Figure S3).

The ANNOVAR annotation was performed to evaluate the genetic variants corresponding to different genomic locations and their functional impact, as detailed in Table 1. The number of non-synonymous sites was observed to be higher than that of synonymous sites, with a nonsyn/syn ratio of 1.16. This ratio is higher than the reported overall nonsyn/syn ratio for germline variants in South Asian populations (1000 Genomes Project Consortium et al., 2015). For normalization, variants within the targeted genomic regions studied in this research were a subset from 1000 Genomes PJL (Punjabi Lahori, Pakistan) individuals, and nonsyn/syn ratio was determined. The PJL healthy individuals showed a ratio of 0.88. The nonsyn/syn ratio in targeted regions was higher in the present study AML cases than in healthy individuals due to the higher proportion of novel non-synonymous variants in the patients, which is persistent with previous reports (Liu et al., 2012).

**TABLE 1:** Total variants pertaining to various genomic regions and their functional distribution.

Genomic region	No. of variants
Exonic	146
Intronic	133
Upstream	0
Downstream	3
UTR5	2
UTR3	9
**Functional Impact**	
Non-synonymous	71
Synonymous	61
Stop-gain	3
Splicing	2
Frameshift insertion	3
Frameshift deletion	2
Non-frameshift insertion	2
Non-frameshift deletion	3

### The Landscape of Somatic Mutations

**FIGURE 2:**
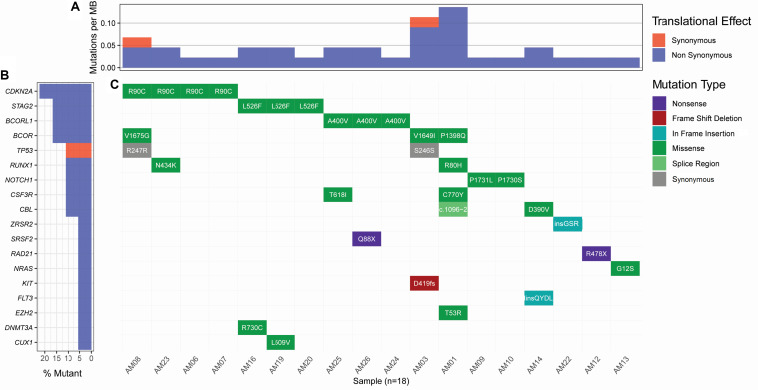
Landscape of somatic mutations in AML. All the non-silent and synonymous somatic mutations are shown here. **(A)** The numbers of non-synonymous mutations are dominantly higher than synonymous mutations at the sequenced targeted regions. **(B)** Percent of samples with the somatic mutation in a gene. **(C)** Detail of somatic mutations in AML cases.

Considering the variants with allelic fraction less than half of the %circulating blasts in each case, there were 38 somatic mutations as a whole, including 31 SNVs and 7 insertions/deletions (1.46 mutation/case). These somatic variations comprised 23 non-silent mutations including 18 non-synonymous SNVs, 2 stop-gain SNVs, 1 splicing SNV, and 2 frameshift deletions, and 17 silent mutations including 2 synonymous SNVs, 2 downstream SNVs, 1 3′untranslated region SNV, 7 intronic SNVs, 2 non-frameshift insertions, and 1 non-frameshift deletion (Figure 2 and Supplementary Table S2). Further, it was observed that some cases contained higher number of somatic mutations in different genes. A Kruskal-Wallis test and post hoc Dunn test of multiple comparisons among all the cases showed a significantly higher number of somatic mutations in two cases, AM01 and AM03 (*p* < 0.01 after multiple corrections).

Strikingly, three recurrent non-silent, and five recurrent silent somatic mutations were observed in more than one case. The non-silent recurrent somatic mutations included a non-synonymous SNV in *CDKN2A* (p.R90C) in four cases, a non-synonymous SNV in *STAG2* (p.L526F) in three cases, and a non-synonymous SNV in *BCORL1* (p.A400V) in three cases. Notably, p.L526F(*STAG2*) and p.A400V(*BCORL1*) affected all the transcripts of respective genes, whereas p.R90C(*CDKN2A*) affected only one out of six transcripts. The VAF of p.R90C (*CDKN2A*) was double in one case AM21 (VAF 0.214) than in the other three cases who carried similar burden of this variant (VAF 0.084–0.105). The mutational burden of two other recurrent mutations, i.e., p.L526F(*STAG2*) and p.A400V(*BCORL1*), was similar among the cases, i.e., 0.102–0.111 and 0.08–0.106, respectively. Further, it was noted that five genes, *CSF3R*, *NOTCH1*, *CBL*, *RUNX1*, and *BCOR*, were found to have independent non-silent mutational events in two cases each, with VAF 0.098–0.108, 0.158–0.278, 0.1448–0.203, 0.159–0.25, and 0.135–0.167, respectively. Two genes, *KIT* and *EZH2*, had two coexisting mutations each (VAF 0.418–0.42 and 0.043–0.045, respectively) in the same cases (AM03 and AM01, respectively), affecting all the transcripts of their genes.

Curation of somatic mutations in COSMIC database highlighted 10 mutational events not observed in the database, whereas four mutations had been cataloged with a different variation type at the sites than observed in this study (detailed in Supplementary Table S2). The non-cataloged 10 mutations also included the stop-gain SNV (p.Q88X) in *SRSF2*, affecting all two transcripts of this gene, and the two recurrent non-synonymous SNVs (p.L526F of *STAG2* and p.A400V of *BCORL1*). Filtration of somatic mutations with ClinVar database highlighted three pathogenic SNVs already associated with hematological disorders. We also assessed conservation status of the non-silent variants sites using PhyloP conservation scores of non-neutral substitution rates based on alignment with 100 vertebrates (Pollard et al., 2010). This analysis revealed 13 mutations in comparatively high conserved regions (PhyloP score >4), 5 mutations in moderately conserved regions (4 ≤ PhyloP score ≥1), and 5 mutations in non-conserved regions (PhyloP < 1) of proteins. Among the recurrent non-silent mutations, p.L526F was observed in the highly conserved region of *STAG2*, suggesting its more profound deleterious effect; p.A400V occurred in moderately conserved region of *BCORL1*, whereas p.R90C occurred in the non-conserved region of *CDKN2A*. The assessment of somatic mutations for potentially driver role through ParsSNP tool highlighted a driver mutation p.G12S in *NRAS* (rs121913250), which is well characterized and already reported recurrently in COSMIC database (COSV54736621 and COSM563).

### Co-existence of Somatic Mutations

**FIGURE 3:**
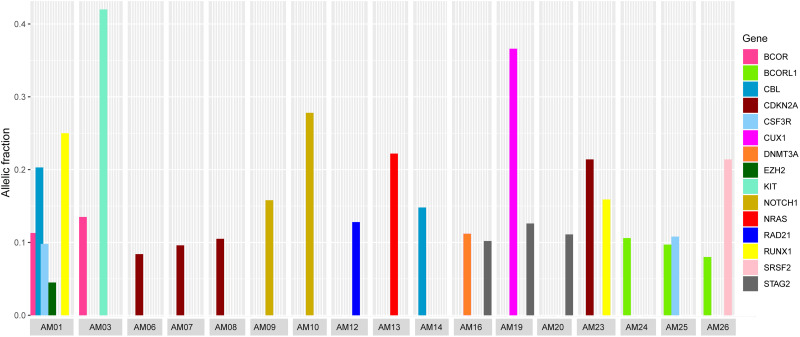
Comparison of variants allelic fraction (VAF) of co-existing non-silent somatic mutations in various cases. The mutations in *RUNX1* and *CBL* in AM01, the *KIT* mutation in AM03, the *CUX1* mutation in AM19, and *SRSF2* mutation in AM26 originated more likely in founding clones, followed by mutations in other genes in subclones.

It was also noted that seven cases carried more than one non-silent variants; however, the genes harboring co-existing mutations were different among all the cases. The difference in VAF of co-existing mutations gave a clue to define the clonal composition, i.e., a founding clone (the clone with the highest VAF values) and the subclone (Figure 3). In AM01, the novel p.R80H mutation in the conserved region of Runt-related transcription factor 1 (*RUNX1*), might be the somatic event (VAF 0.25) followed by disrupting splice site (c.1096-2) in Cbl proto-oncogene (*CBL*) (VAF 0.203) in founding clones leading to the abnormal proliferation of hematopoietic stem cells. Analysis of protein interaction between RUNX1 and CBL through STRING database revealed no interaction between these two proteins, indicating independent mutational events. Previously, a different mutation, p.R80A, at same position of *RUNX1*, has been shown to strongly reduce its binding with DNA (Bravo et al., 2001). In AM03, the co-existing p.D419fs and p.R420fs deletions in *KIT* (VAF 0.418 and 0.42) originated more probably in founding clone, prior to the p.V1649I of *BCOR* (0.135) in subclone. In AM19, the novel p.L509V mutation in *CUX1* (VAF 0.366) might be originated in founding clone, followed by p.L526F of *STAG2* (0.126) in subclone; in AM26, the p.Q88X in *SRSF2* (VAF 0.214) in founding clone and p.A400V in *BCORL1* (VAF 0.08) in subclone. The co-existing somatic mutations in three other cases, AM16 [p.R730C of *DNMT3A* (VAF 0.112) and p.L526F of *STAG2* (VAF 0.102)], AM23 [p.R90C of *CDKN2A* (VAF 0.214) and p.N434K of *RUNX1* (VAF 0.159)], and AM25 [p.T618I of *CSF3R* (VAF 0.108) and p.A400V of *BCORL1* (VAF 0.097)], more probably originated in the same clones. It was noteworthy that all the coexisting mutational events happened in genes belonging to different biological functional categories previously described in myeloid leukemias (Cancer Genome Atlas Research Network et al., 2013), indicating that different underlying processes were involved in the pathophysiology of AML in this cohort.

### Germline Mutation Predisposition

In addition to the somatic mutations, the predisposition due to germline mutations was also assessed. For this, the germline variants with ClinVar pathogenic/likely pathogenic significance, producing a stop-gain or stop-loss site, disrupting splicing sites, frameshift insertions, or deletions, and non-synonymous alterations predicted as deleterious by SIFT and Polyphen2 tools, were brought into subsequent analysis, as described previously (Bertelsen et al., 2019). This analysis prioritized 18 germline variants pertaining to 15 genes including 13 non-synonymous SNVs, a stop-gain SNV, a splice-site SNV, and 3 frameshift insertions (Supplementary Table S3). Two recurrent mutations, p.T358P in *GATA2* affecting all three transcripts, and p.L258fs insertion in *NPM1* affecting two out of seven transcripts, were observed in two non-related cases each. Further, *WT1* was observed recruiting three independent germline mutations in three different unrelated cases. Filtration with ClinVar database highlighted six non-synonymous pathogenic SNVs and a frameshift pathogenic insertion already associated with hematological neoplasms. The germline variants were filtered with COSMIC database, which revealed six novel variants not cataloged in this database. The assessment of PhyloP scores revealed 11 mutations in comparatively high conserved regions, 2 mutations in moderately conserved regions, and 5 mutations in non-conserved regions of respective proteins. The variants affecting highly conserved regions included the recurrent p.T358P in *GATA2*, protein truncating p.R441X in *WT1*, and splicing c.418+1 in *PHF6*. Exploration of protein truncating p.R441X (rs121907909) in Ensembl genome browser^2^ revealed that it affects eight protein coding transcripts introducing a premature stop codon, whereas two protein coding transcripts are protected through NMD pathway (Supplementary Table S4).

To explore the role of identified genetic variants in drug response, pharmGKB database was searched. This analysis showed a missense SNV rs1042522 (G > C) in *TP53*, with GG and GC genotypes in 19 (73%) cases. These genotypes have been found to show decreased response to cisplatin, paclitaxel, capecitabine, and oxaliplatin anti-cancer drugs as compared to the CC genotype.

## Discussion

Next-generation sequencing analysis of myeloid neoplasm including AML and other related disorders has yielded several significant advances in the identification of diagnostic, prognostic, and therapeutic markers for these disorders (Arber et al., 2016; Papaemmanuil et al., 2016). This study was designed to screen AML patients in a clinical diagnostic setup by using a specifically designed myeloid sequencing panel and provides clinico-pathological significance of identified deleterious/non-silent mutations in the Pakistani population. We identified 293 variants including single-nucleotide variants, and small insertions and deletions in coding as well as in non-coding regions in a small cohort of 26 AML patients. Sequence variants not observed in ClinVar, dbSNP, and gnomAD were considered as novel variants. The pathogenicity of sequence variants with a global minor allele frequency (GMAF) of <0.1 was assessed by using several *in silico* bioinformatics tools and the variants were classified according to the ACMG criteria (Richards et al., 2015). The deleterious impact of non-synonymous variants was assessed with SIFT, Polyphen2, and CADD, as described previously (Shakeel et al., 2018). Although variants were not functionally validated using any in vitro system, *in silico* analyses have generated strong and convincing scores that suggest the possible pathogenicity of the identified variants in respective cases. To the best of our knowledge, this is the first study to report genetic variations in myeloid malignancies from this South Asian population using NGS technology.

**FIGURE 4:**
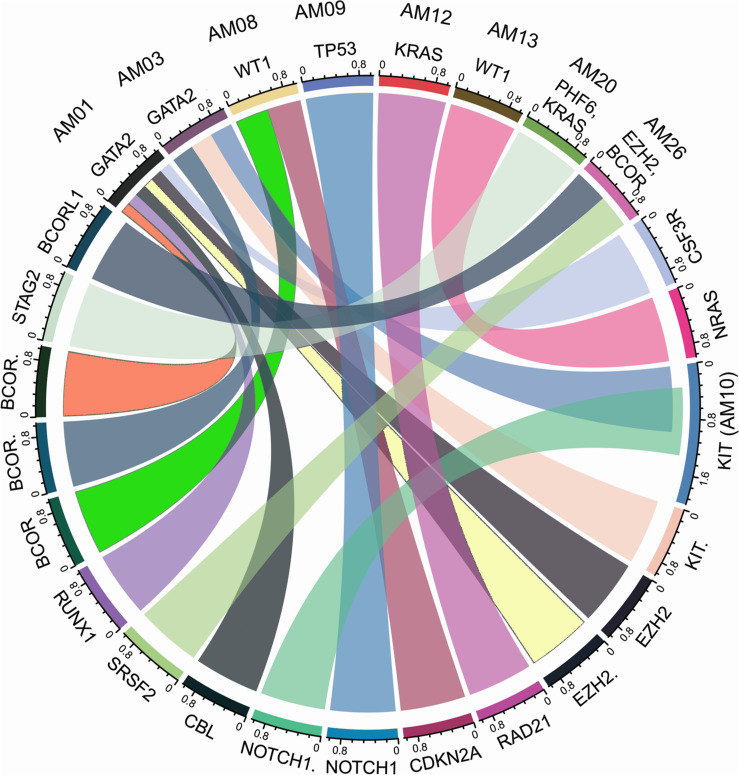
Co-existence of prioritized/pathogenic germline variants with the non-silent somatic mutations in cases with both germline and somatic events. The names of cases are mentioned above the genes with germline mutations.

The higher nonsyn/syn ratio in AML cohort represents higher mutation rate and/or positive selection on non-synonymous sites, as indicated in various cancers previously (Greenman et al., 2007; Pengyuan et al., 2012). By applying the multi-tool prioritization approach, we were able to find at least one pathogenic/deleterious non-silent somatic or predisposing germline mutation in 23 of the 26 cases (88.46%), where 9 cases had both the somatic and germline mutations, 8 cases had somatic mutation only, and 6 cases had germline mutation only. In order to explore possible biological relationship between a predisposing germline mutation and somatic mutational events in the cases having coexisting germline and somatic mutations, a circos plot was constructed, which revealed that the two cases with germline non-synonymous mutation p.T358P in *GATA2* (AM01 and AM03) had higher number of non-silent somatic mutations (Figure 4). *GATA2* encodes an endothelial transcription factor GATA-2 that plays an essential role in gene regulation during vascular development and hematopoietic differentiation. The observed mutation is within the highly conserved region of GATA2 (a zinc finger domain). Although this mutation is novel and not cataloged in dbSNP or COSMIC databases, a p.R361P change near the observed p.T358P mutation in the same zinc finger domain has been shown to be associated with Emberger syndrome (lymphedema with predisposition to AML) (Ostergaard et al., 2011). Further, search in STRING database showed experimental and curated pathway interaction between GATA2 and RUNX1, the two mutated genes in AM01 (Supplementary Figure S4). It has been shown previously through Chip-seq analysis that there is concurrent binding of GATA2 and RUNX1 along with GATA1, FLI1, and SCL transcription factors on promoters of a set of genes, e.g., *CEBPA*, which are highly enriched for known regulators of hematopoiesis (Timchenko et al., 1995; Tijssen et al., 2011).

The genetic heterogeneity and the complex interaction among different oncogenic pathways in AML have been focused in previous studies to explore its prognostic significance (Estey, 2014). The most recurrent non-silent somatic mutation p.R90C in cyclin-dependent kinase inhibitor 2A gene (*CDKN2A*) was found in four cases. The observed variant belongs to non-conserved region of *CDKN2A*, yet it has been cataloged in ClinVar associated with hereditary cancer-predisposing syndrome with uncertain significance. The kinase inhibitor arrests cell cycle at G1 and G2 stages and acts as a tumor suppressor (Genecards database, 2019). The arginine-to-cysteine substitution as a result of this variation may hamper its ability to arrest cell cycle, leading to accelerated cell proliferation. Although this variant is ultra-rare (alternate allele frequency of 4.71 × 10^–6^ in the gnomAD database and 8.42 × 10^–6^ in the ExAC database), its recurrence in 15% of our cases suggests its likely prognostic role in AML in this population. The second recurrent non-silent somatic mutation p.L526F (*STAG2*) occurs in a conserved domain of cohesin subunit SA-2, which is a component of the cohesin complex required for the cohesion of sister chromatids after DNA replication. Previously, non-silent mutations at different sites in *STAG2* were found in 1.3% of AML cases (Thol et al., 2014), whereas, in this study, the observed mutation was found in 11.5% cases. The third novel recurrent mutation p.A400V was observed in *BCORL1*, affecting three cases. *BCORL1* encodes BCL6 corepressor like 1 protein, which specifically inhibits gene expression when recruited to promoter regions by sequence-specific DNA-binding proteins such as BCL6 (Pagan et al., 2007). The concurrence of non-silent mutations in genes belonging to different functional categories represents the heterogeneity of AML in this cohort, which is persistent with previous reports (Cancer Genome Atlas Research Network et al., 2013).

The novel non-synonymous somatic mutations p.V1675G, p.V1649I, and p.P1398Q in conserved regions of the BCL6 co-repressor (*BCOR*) were found independently in 11.5% cases (three of this cohort), which is three times higher compared to those reported by Grossmann et al., where *BCOR* gene mutations were identified in 3.8% (10 of 262) of cytogenetically normal (CN) AML cases with poor response (Grossmann et al., 2011). It is noteworthy that *BCOR* also contained a germline deleterious non-synonymous mutation p.S1582G. Strikingly, this mutation was also not found in COSMIC database. Together with the germline mutation, the frequency of *BCOR* non-silent/deleterious mutations becomes 15.4% (four cases). This depicts *BCOR* as a high-risk gene in the South Asian population. The *BCOR* encoded protein, BCL6 co-repressor, is a component of a variant Polycomb group repressive complex 1, and has the ability to specifically repress gene transcription when recruited to promoter regions by sequence-specific DNA-binding proteins such as BCL6 and MLLT3 (Huynh et al., 2000; Sanchez et al., 2007). It contributes as a major player in the embryonic differentiation and mesenchymal stem cell function (Wamstad et al., 2008; Fan et al., 2009). Recently, BCOR mutant bone marrow cells showed significantly higher proliferation and differentiation rates with upregulated expression of HOX genes (Cao et al., 2016).

The novel protein truncating somatic mutation in *SRSF2* generates alteration of c.C262T in exon1 of the resulting transcript, leading to the premature termination at p.Q88X, and causing inactivation of the RNA-binding domain (residues 1–101) of the protein. This mutation affects five protein-coding transcripts, whereas two transcripts undergo non-sense-mediated decay. The *SRSF2* is a member of the serine/arginine rich (SR) class of splicing factors involved in both constitutive and alternative mRNA splicing. Previously, dysfunctional SRSF due to sequence variations at p.P95H position have been found to activate aberrant alternative splicing in hematopoietic cells, whereby, having its role in onset of myelodysplastic syndromes (MDS) and AML (Liang et al., 2018; Masaki et al., 2019). In this context, the truncating mutation observed in this study would more likely result in non-functional SRSF, which would lead to malignancy due to hampered alternative splicing in hematopoietic cells. This study reports a first truncating mutation in the SRSF2 RNA-binding domain in an AML case. The other stop-gain somatic mutation in *RAD21*, causing a protein truncation at p.R478X, affects two protein coding transcripts. This variation has been cataloged in the COSMIC database with four recurrences (COSM1735718) associated with AML. The third and germline stop-gain mutation in *WT1* causes p.R441X truncation. Previously, the protein truncating variations in *WT1* have been shown to attenuate the TP53-induced DNA damage response in T-cell acute lymphoblastic leukemia (Bordin et al., 2018).

## Conclusion

In conclusion, this is the first report of a comprehensive analysis of somatic as well as germline mutations in AML from Pakistan using next-generation DNA sequencing technology. Our data strongly support and extend the spectrum of detrimental mutations identified in previous studies employing targeted resequencing approach for the diagnosis of AML. This study also highlights the usefulness of panel sequencing in cases where prognosis becomes challenging. The small cohort size and retrospective nature, i.e., sample collection from a single medical center, are the limiting factors of the study. Furthermore, the novel findings of this preliminary study require validation in a larger cohort with different time scales. Nevertheless, the findings provide an assessment of predisposing detrimental mutations of AML in this region and its utility in clinical settings.

## Data Availability Statement

The raw datasets presented in this article have been deposited in BioProject – accession PRJNA627793.

## Ethics Statement

The studies involving human participants were reviewed and approved by Research Ethics Committee and Review Board of NIBD. Written informed consent to participate in this study was provided by the participants’ legal guardian/next of kin.

## Author Contributions

SSh: study design and execution, and manuscript writing. MSh: data analysis, result interpretation, and manuscript writing. SSi: clinical examination and evaluation of AML patients. SA: library preparation for myeloid sequencing panel. MSo: DNA extraction and quantification. AA: review of manuscript. IK and TS: involving in study design, patient recruitment, review of manuscript, and supervision throughout the study.

## Conflict of Interest

The authors declare that the research was conducted in the absence of any commercial or financial relationships that could be construed as a potential conflict of interest.

## Footnotes

1 http://picard.sourceforge.net

2 www.ensembl.org/index.html

## Supplementary Material

FIGURE S1 Workflow for DNA library preparation using Illumina TruSight myeloid sequencing panel.

FIGURE S2 Electropherograms of Sanger sequencing of identified mutations in AML cases.

FIGURE S3 Density plot to show the distribution/spectrum of variants allelic fraction (VAF) of all variants across the 26 AML patients of this study. The perpendicular at 0.51 allele fraction represents the median percentage of circulating blasts cells across all cases.

FIGURE S4 Protein-protein interaction between GATA2 and the proteins with somatic mutations in AM01.

TABLE S1 Clinical features of 26 AML cases.

TABLE S2 SOMATIC Mutations.

TABLE S3 Germline Mutations in AML.

TABLE S4 Effect of stopgain and frameshift variants (germline and somatic) on transcripts.
