# Supplementary figures and images for: Novel Genetic Variations in Acute Myeloid Leukemia in Pakistani Population
Source: Front Genet. 2020 Jun 23;11:560. doi: 10.3389/fgene.2020.00560 (PMC7324646; doi:10.3389/fgene.2020.00560)

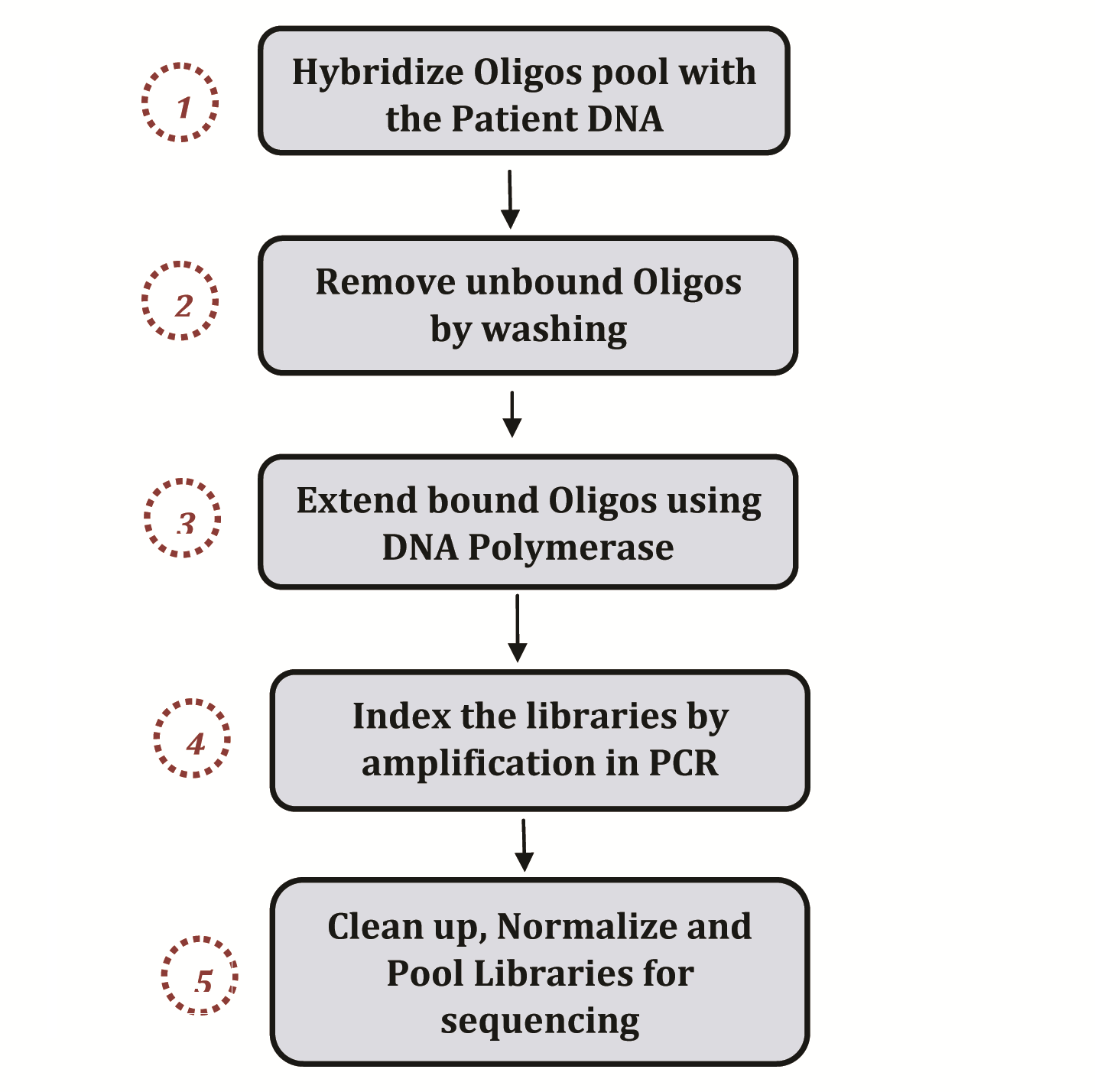

Supplement: FIGURE S1 — Workflow for DNA library preparation using Illumina TruSight myeloid sequencing panel. [file Image_1.TIF]

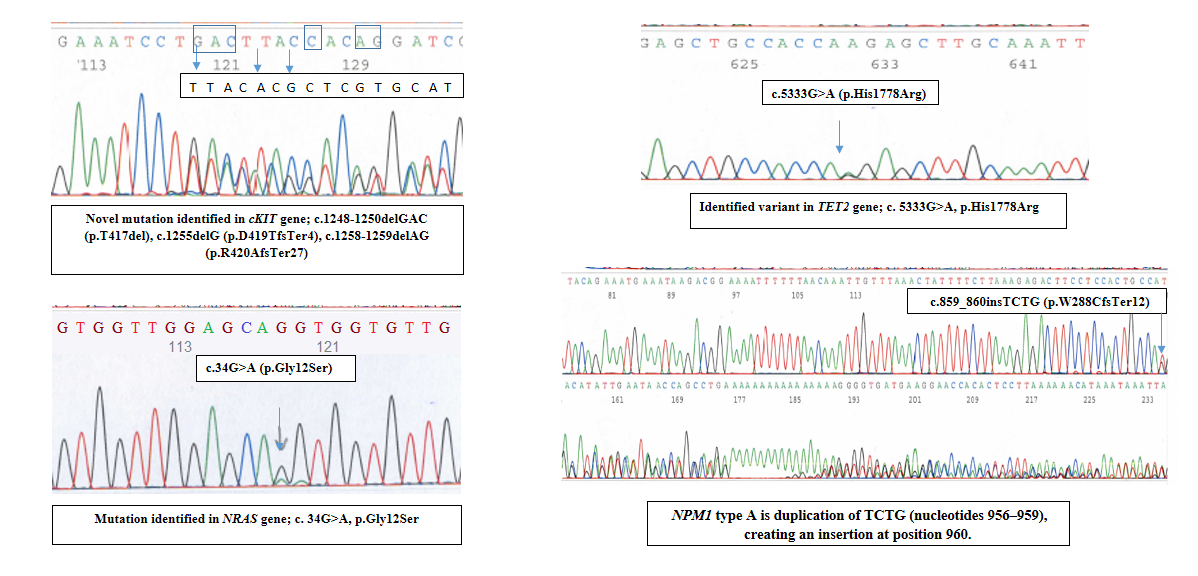

Supplement: FIGURE S2 — Electropherograms of Sanger sequencing of identified mutations in AML cases. [file Image_2.TIF]

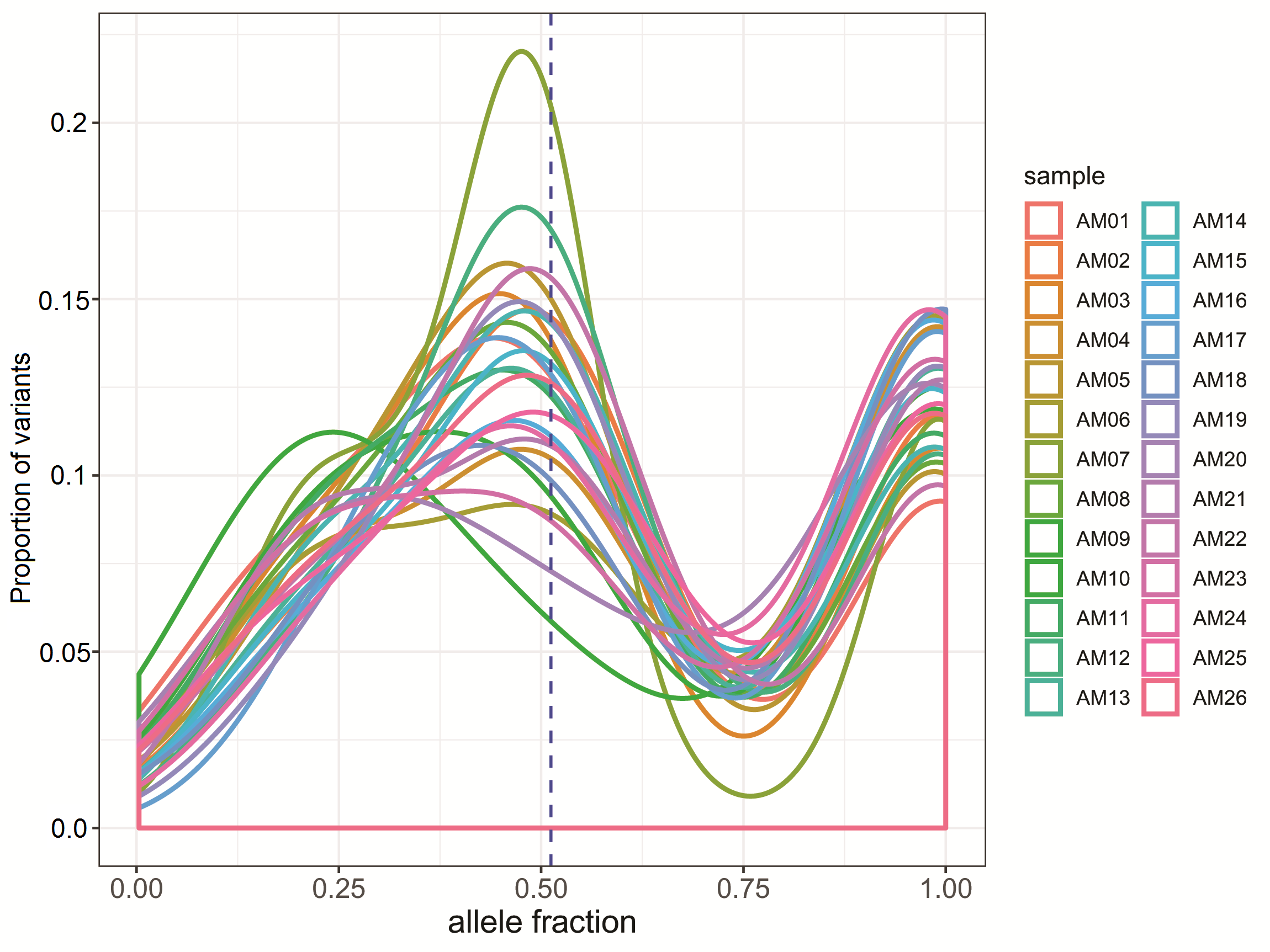

Supplement: FIGURE S3 — Density plot to show the distribution/spectrum of variants allelic fraction (VAF) of all variants across the 26 AML patients of this study. The perpendicular at 0.51 allele fraction represents the median percentage of circulating blasts cells across all cases. [file Image_3.TIF]

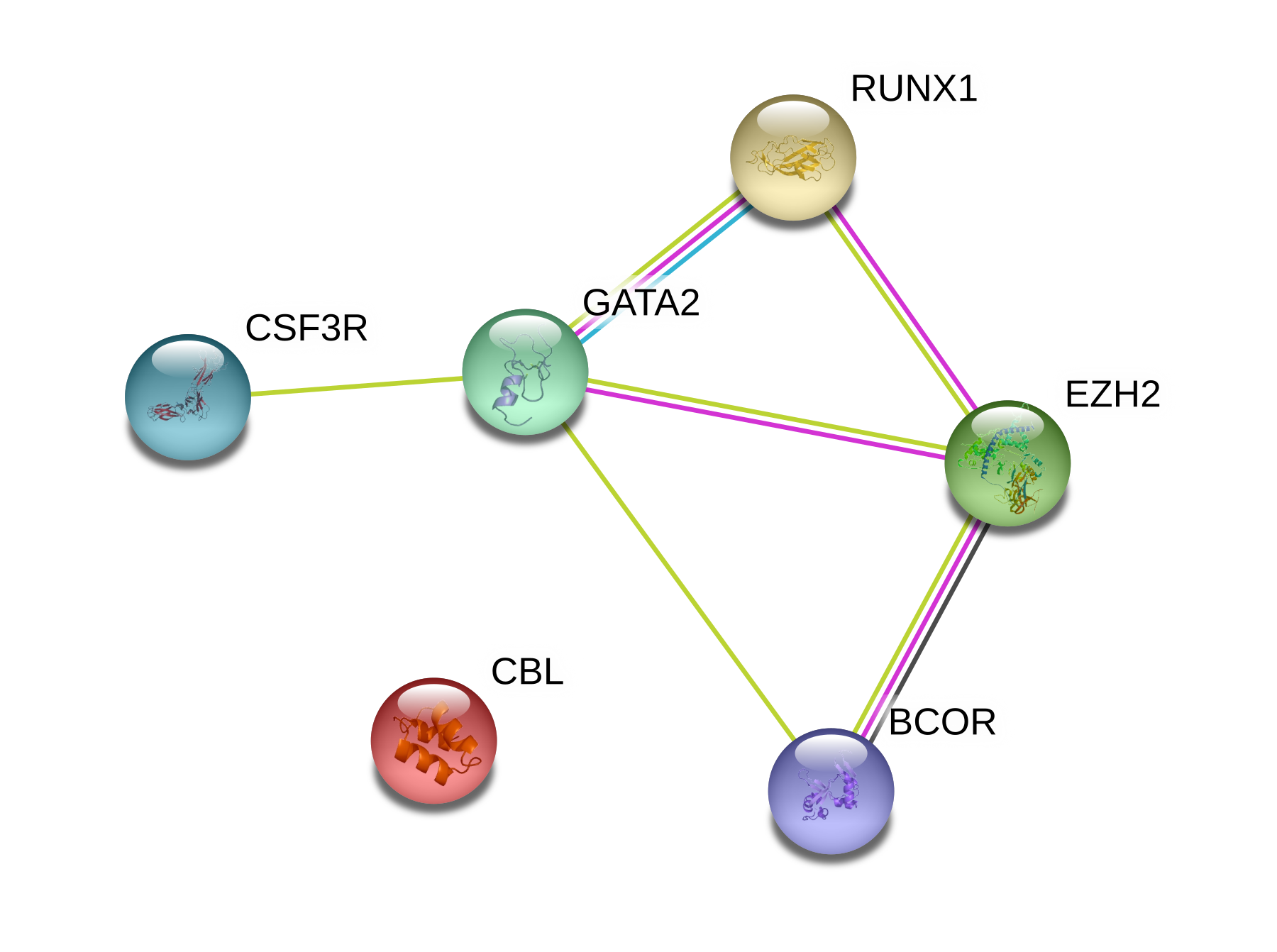

Supplement: FIGURE S4 — Protein-protein interaction between GATA2 and the proteins with somatic mutations in AM01. [file Image_4.TIF]
